# Viscoelastic Properties of Acellular Matrices of Porcine Esophageal Mucosa and Comparison with Acellular Matrices of Porcine Small Intestine Submucosa and Bovine Pericardium

**DOI:** 10.3390/ma17010134

**Published:** 2023-12-27

**Authors:** Sergio Estrada Mira, María Inmaculada García-Briega, José Luis Gómez Ribelles, Luz M. Restrepo Munera

**Affiliations:** 1 Tissue Engineering and Cells Therapy Group (GITTC), School of Medicine, University of Antioquia, Medellin 050010, Colombia; sergio.estrada@udea.edu.co (S.E.M.); grupoingenieriadetejidos@udea.edu.co (L.M.R.M.); 2 Cell Therapy and Biobank, Alma Mater Hospital of Antioquia, University of Antioquia, Medellin 050010, Colombia; 3 Centre for Biomaterials and Tissue Engineering (CBIT), Universitat Politècnica de València, 46022 Valencia, Spain; inmagarciabriega@gmail.com; 4 Centro de Investigación Biomédica en Red de Bioingeniería Biomateriales y Nanomedicina (CIBER-BBN), Instituto de Salud Carlos III, 28029 Madrid, Spain

**Keywords:** decellularized tissue, porcine esophageal mucosa, viscoelastic properties, compliance, elastic modulus, shear modulus

## Abstract

The aim of this study was to compare the viscoelastic properties of a decellularized mesh from the porcine esophagus, prepared by our group, with two commercial acellular tissues derived from porcine small intestine submucosa and bovine pericardium for use in medical devices. The tissues’ viscoelastic properties were characterized by creep tests in tension, applying the load in the direction of the fibers or the transverse direction, and also by dynamic-shear mechanical tests between parallel plates or in tension at frequencies between 0.1 and 35 Hz. All the tests were performed in triplicate at a constant temperature of 37 °C immersed in distilled water. The tissues’ surface and cross-sectional microstructure were observed by scanning electron microscopy (SEM) to characterize the orientation of the fibers. The matrices of the porcine esophagus present an elastic modulus in the order of 60 MPa when loaded in the longitudinal direction while those of the porcine intestine submucosa and bovine pericardium have an elastic modulus below 5 MPa. Nevertheless, the shear modulus of bovine pericardium nearly triplicates that of the esophageal matrix. The viscoelasticity of decellularized esophageal mucosa is characterized by a fast change in the creep compliance with time. The slope of the creep curve in the double logarithmic plot is twice that of the control samples. These results are consistent with the microstructure observed under electron microscopy regarding the orientation of the fibers that make up the matrices.

## 1. Introduction

Developing devices based on an extracellular matrix (ECM) for either scaffolds for cell culture or surgical biological meshes requires them to have the necessary biomechanical characteristics to sustain the stresses they will be subjected to [1,2,3].

Biological tissues are dynamic structures that serve essential functions within the human body. They are subjected to various mechanical stresses and strains over time, and their mechanical behavior plays a critical role in their physiological functions and response to injuries. Conventionally, mechanical testing has often emphasized the study of rupture behavior, which provides insights into the point at which a material fails under load. While rupture behavior is undoubtedly significant, it offers only a limited perspective on tissue mechanics. A more comprehensive understanding can be achieved by assessing the viscoelastic properties in acellular tissues [4].

The processes used to obtain biomaterials based on human or animal tissues change their general biological properties, including improving their biocompatibility (especially eliminating the cellular component), although this process may involve matrix degradation and the loss of some structural proteins. All the processes used to obtain ECM-based biomaterials aim to achieve complete decellularization while preserving the structural proteins responsible for the biological and mechanical properties [5,6,7,8,9].

Type I collagen is the main component of the extracellular matrix of many tissues. This protein has a deformation at break of between 10 and 20% [10,11]. Another important protein in ECM is elastin, which provides its elastic characteristics, or the ability to recover its original shape after applying a defined force without suffering ruptures or permanent deformation. The viscoelastic properties of the extracellular matrix, which includes the vital contributions of both collagen and elastin, are further enhanced by the presence of proteoglycans. The extracellular matrix is a complex network of proteins and carbohydrates that provides structural support to various biological tissues.

Collagen and elastin, as mentioned earlier, offer tensile strength and elasticity to the matrix. However, proteoglycans play a unique role in enhancing the viscoelastic behavior of these tissues. Proteoglycans are large molecules consisting of a core protein and long chains of carbohydrates called glycosaminoglycans. These molecules occupy spaces within the matrix and interact with water [12].

Proteoglycans have a high affinity for water molecules, and their presence creates a hydrated gel-like environment within the extracellular matrix. This gel structure, along with the collagen and elastin fibers, contributes to the viscoelastic properties of the tissue.

The interaction between proteoglycans and water allows the matrix to resist compressive forces while also providing a mechanism for tissue deformation and recoil over time. This unique combination of properties ensures that biological tissues can withstand mechanical stresses, maintain their shape, and return to their original state, making proteoglycans a crucial component in the dynamic viscoelastic behavior of the extracellular matrix. These properties are essential in various physiological processes, including tissue flexibility, load-bearing, and response to mechanical forces in the body. The combination of all of them is responsible for the viscoelastic behavior observed in many biological tissues [13,14].

The variations in the mechanical properties of the different tissues depend on the ratio of their elastic components, such as elastin, and their stiff components, such as fibrillar collagen. For example, the thoracic aorta contains close to 50% of its dry weight in elastin to support the continuous dilation and contraction cycles during systole and diastole at least 60 times per minute throughout the life of an individual, while the tendons are mainly formed by type I collagen since they require greater tensile strength and less elasticity to efficiently transmit the force generated by the muscles to move the limbs [15,16,17,18,19,20,21].

However, the biomechanical behavior is not only determined by the proportions of ECM components, since the isotropy or anisotropy of these components also determine the global behavior of the tissue. The elastin and collagen in the arteries are arranged circumferentially, forming lamellae, while the collagen in the tendons is aligned longitudinally and parallel to the direction in which muscle traction is exerted [22].

As ECM is a polymeric material, its response mechanisms to movement or deformation are characterized by the time delay of the response (deformation or stress) concerning the action applied to it (stress or deformation, respectively). The success or failure of the in vivo implantation of a decellularized tissue may depend on its ability to precisely mimic the mechanical response of natural tissue. This not only reproduces the tissue’s stiffness but also its viscoelastic response. Effort has been made to determine the role of the tissue microstructure, its composition, and the packing and ordering of its fibers in the viscoelastic response, and although some models have been proposed to describe it, the problem continues to be a challenge [23,24,25,26,27,28].

In the present study, the viscoelastic response of a decellularized mesh from the porcine esophagus (PEM) prepared by our group [29] was characterized taking two commercial acellular medical devices derived from porcine small intestine submucosa (SIS) and bovine pericardium (BP) as references.

## 2. Materials and Methods

### 2.1. Preparation of Extracellular Matrix from Porcine Esophagus

The protocols used to obtain and process the animal tissue were approved by the Institutional Committee for the Care and Use of Experimental animals (CICUA) of the University of Antioquia, num 101/12 February 2016.

Esophagi were obtained from pigs aged between 12 and 16 weeks destined for human consumption in a local market. Between 20 and 25 cm of the thoracic portion were taken from the esophagi and the mucosa was dissected manually. The mucosa was washed with a sterile saline solution (Corpaul, Col, Antioquia, Colombia) to remove debris, vacuum packed in plastic bags, and frozen for at least 24 h at −20 °C before decellularization. After thawing, the membranes were washed in 250 mL of phosphate-buffered saline (PBS) (Sigma, Burlington, MA, USA) for 3 h. The PBS was then removed and 250 mL of 1% triton X-100 (Sigma) was added for 24 h, washed with 250 mL of PBS for 2 h, a new cycle was repeated with 250 mL of triton X-100 at the same concentration for 24 h, washed again with PBS for 1 h before changing the PBS for sodium dodecyl sulfate SDS (Sigma) at 1% for 24 h, washed again with 250 mL of 1% PBS for one hour, and the PBS was changed again for 1% SDS for another 24 h.

All the above steps were carried out under magnetic stirring at 300 rpm and 4 °C. The matrices were then frozen under vacuum at −20 °C for 24 h, with one group in a single layer (PEMs) and another in two layers perpendicular to each other (PEM-D or Double PEM), and vacuum dried at a temperature of 50° C. Figure S1 in the Supplementary Material shows the absence of cells in decellularized tissue [29].

Commercial submucosal matrices from the acellular small intestine (SIS) were acquired, measuring 3 cm × 5 cm (3-Biomat, Bogotá, Colombia), and bovine pericardium (B shows the absence of cells in the decP) measuring 5 cm× 10 cm (Porites, Medellín, Colombia), for comparison with the matrices’ viscoelastic properties.

The thickness of the samples was determined by measuring their cross-section using electron microscopy, and the average thickness of the evaluated samples was as follows: PER 586 µm (SD 52.59); SIS 417 µm (SD 137.8); single PEM 183.5 µm (SD 14.32); and double PEM 250.6 µm (SD 23.19).

### 2.2. Scanning Electron Microscopy

Longitudinal and transversal decellularized tissue samples were examined by SEM. The longest side of the commercial sample was labeled as longitudinal, while the PEM sample in the cephalocaudal direction of the esophagus was considered to be longitudinal and transversal in the coronal direction.

The samples were fixed with 10% buffered formaldehyde (Merck, Darmstadt, Germany) for 24 h, after which the formaldehyde was removed and 2.5% glutaraldehyde (Merck) was added, left in this solution for 2 h, and dehydrated by a successive series of 30%, 50%, 70%, 90%, 95%, and 99% ethanol. The samples were critical-point dried (Sandri PVT 3D Tousimis, Rockville, MD, USA) at 31 °C and 1072 PSI, fixed on graphite tape, and sputtered with gold (Denton Vacuum Desk IV, Moorestown, NJ, USA.). The samples were evaluated in a scanning electron microscope (Jeol JSM 6490 LV, Peabody, MA, USA) under vacuum to obtain high-resolution images. A secondary electron detector was used to evaluate the samples’ morphology and topography.

### 2.3. Creep Tests

Tensile creep tests were performed (Seiko Exstar TMA ss6000 dilatometer, Chiba, Japan) with 1 cm × 5 mm specimens of the test areas and variable thickness, according to the sample studied, in triplicate in both the transverse and longitudinal directions and immersed in a thermoregulated distilled water bath at 37 °C. The test samples were mounted between two steel plates secured by screws to prevent slipping (according to the equipment manufacturer’s instruction). The following protocol was used for all the tests: preloading with a load ramp from 0 to 10 mN, at 5 mN per minute, a waiting time of 5 min with a load of 10 mN, a load from 10 to 500 mN at a speed of 100,000 mN per minute, and keeping the load at 500 mN for 60 min, the discharge was performed from 500 to 10 mN and at a speed of 100,000 mN per minute, while deformation was recorded at 10 mN for 15 min. The discharge was evaluated for 90 min in a sample from each group. To analyze the results, compliance was calculated: J(t) = γ (t)/σ, which was represented versus the logarithm of time in seconds (compliance is a function of time, denoted by J(t), the ratio of the deformation or strain (γ) at that time (t), and the applied stress (σ)).

### 2.4. Rheological Tests between Parallel Plates

The samples’ rheological behavior was studied by a dynamic oscillatory rheometer between 12 mm diameter parallel plates (TA Instruments, DHR 2, New Castle, DE, USA) in a humidified chamber with the lower plate at a constant temperature of 37 °C. First, 12 mm diameter circumferential samples were cut from each matrix and hydrated in distilled water for 15 min before testing. The tests were in triplicate, using an automatic approximation up to a resistance of 0.3 N. A stress sweep test was performed to determine the linear viscoelastic region at a frequency of 1 Hz with a strain of between 0.01 and 15%. The frequency sweep test was performed with a deformation of 0.3% and a frequency range between 0.1 and 20 Hz. The results were analyzed on TA Instruments TRIOS software V3.1.0.3538.

### 2.5. Dynamic-Mechanical Measurements (DMA) in Tension Mode

Dynamic-mechanical tests (DMA 8000, Perkin Elmer, Waltham, MA, USA) were carried out on 1 cm long × between 5 and 8 mm wide areas and variable thickness in triplicate, and both transverse and longitudinal directions with the samples immersed in a thermoregulated distilled water bath at a constant temperature of 37 °C. A frequency scan was performed between 0.1 Hz and 35 Hz. 

### 2.6. Statistics

All experiments were performed with three biological replicates. The results are given as the mean ± standard deviation (SD). The normality of the different samples was checked using the Shapiro–Wilk normality test with an alpha value of 0.05. For comparisons of two single groups of data, unpaired T-Student tests (*p*-value = 0.05) were completed. An ordinary one-way ANOVA test (*p*-value = 0.05) was used for three or more groups to perform multiple comparisons between the column means when the normality test was passed. If the normality test was not passed, then the non-parametric Kruskal–Wallis test was used to compare this non-normal sample with other normal samples (*p*-value = 0.05) to perform multiple comparisons between the column means. GraphPad Prism 8 software (GraphPad Software, La Jolla, CA, USA) was used for statistical analysis. Differences among the groups are stated as *p* ≤ 0.05 (*), *p* ≤ 0.01 (**), *p* ≤ 0.001 (***), and *p* ≤ 0.0001 (****) in normal samples, and *p* ≤ 0.05 (#) in non-normal ones. 

## 3. Results

### 3.1. Microestructure

**Figure 1:**
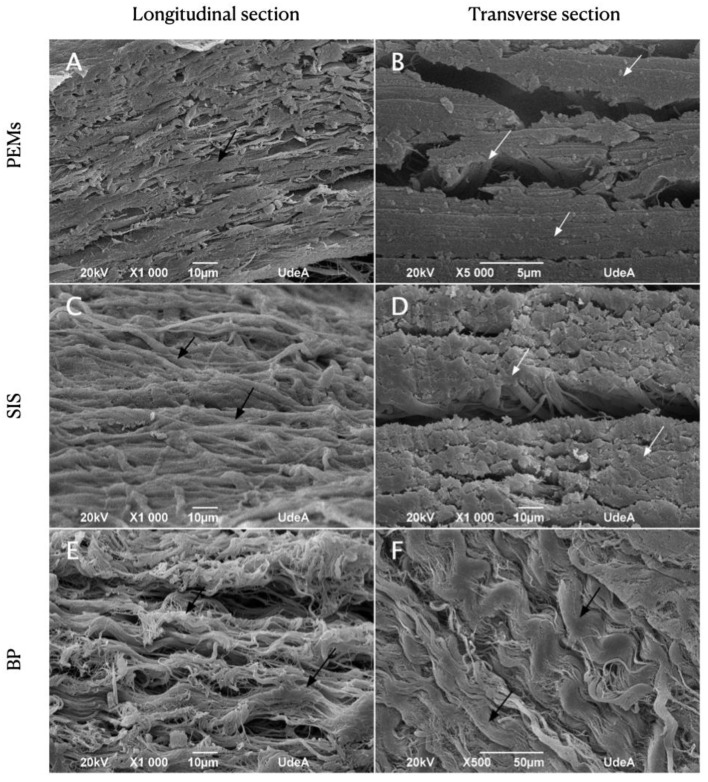
Longitudinal (**A**,**C**,**E**) and transversal (**B**,**D**,**F**) sections of PEM SEM images (**A**,**B**), SIS (**C**,**D**), and BP (**E**,**F**). Collagen fibers longitudinally oriented (black arrows) and cut transversely (white arrows) can be seen.

SEM analysis of the matrices in both longitudinal and transverse directions revealed that the collagen fibers in the PEMs were predominantly oriented longitudinally (Figure 1A,B). Notably, Figure 1B (highlighted by white arrows) provides an axial view of collagen fibers within a transverse cross-section. This specific alignment of collagen fibers in the PEMs is significant because it can influence the material’s mechanical properties and its suitability for specific applications. It may facilitate better load-bearing characteristics in the direction of longitudinal alignment and impact the tissue engineering or regenerative medicine potential of these matrices.

Similarly, in the SIS matrices (Figure 1C,D), collagen fibers exhibited a similar directional alignment to that of the PEMs, primarily running in a singular direction. This consistency in the fiber orientation between the two matrix types may suggest that they share certain structural or functional characteristics. This alignment of collagen fibers, especially if it parallels that of native tissues, can enhance the biomimetic nature of these matrices for tissue repair and regeneration applications.

In contrast, the collagen fibers in the BP matrices (Figure 1E,F) appeared scattered, lacking a predominant orientation either transversely or longitudinally. The lack of a distinct orientation may impact the mechanical behavior and effectiveness of BP matrices for certain applications, potentially making them less ideal for specific tissue engineering needs.

### 3.2. Viscoelastic Response

All three tested materials behaved viscoelastically, as shown by their evolution over time in compliance with the creep tests in tension and also by the frequency dependence of the complex elastic modulus in tension and shear (Young modulus E* = E′ + iE″ and shear modulus G* = G′ + iG″) in the dynamic response tests, as we will see below. 

**Figure 2:**
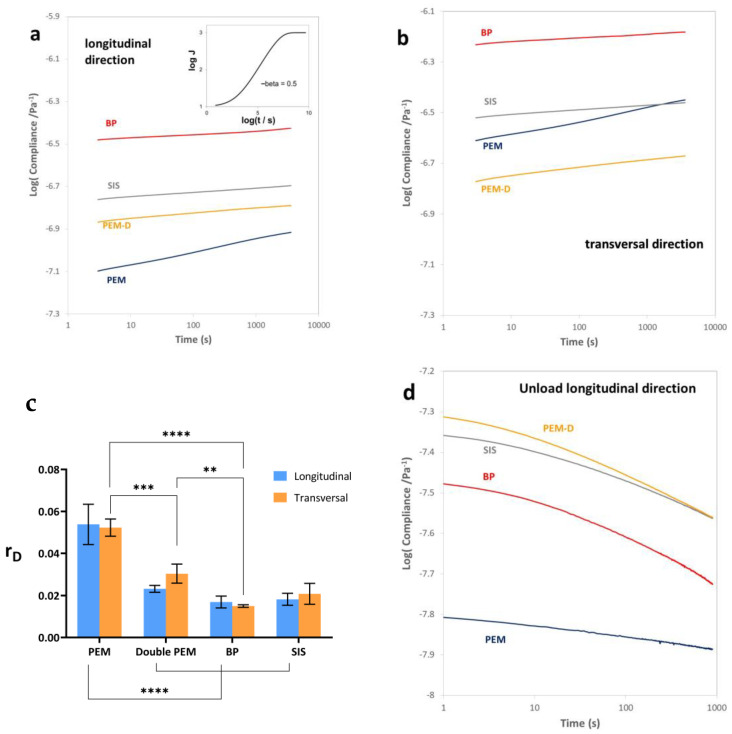
Compliance of the decellularized matrices of porcine esophageal mucosa of one, PEM, or two, PEM-D, layers of small intestine submucosa, SIS, and bovine pericardium, BP measured in creep test in tension mode. (**a**) Measurements in the longitudinal direction. The inset shows a schema of a relaxation process following the KWW equation with an exponent β = 0.5, showing that it covers a time interval of the order of eight decades. (**b**) Measurements in the transverse direction. (**c**) Response rate (r_D)_, and (**d**) unloading measured in the longitudinal direction. Bar diagram shows mean ± standard deviation. The level of statistical significance is shown by the following legend: *p* ≤ 0.01 (**), *p* ≤ 0.001 (***), *p* ≤ 0.0001 (****).

Deformation was measured in the tensile creep test as a function of time both in the loading process, for 3000 s, and in the subsequent unloading. All the samples were subjected to the same tension and the tests were carried out immersed in distilled water at 37 °C. The values of compliance in tension D(t) measured in the longitudinal direction are shown in Figure 2a. All the samples showed a higher deformability in the transverse direction than the longitudinal (Figure 2b). The characteristic behavior of a viscoelastic solid in the log D vs. log t double-logarithmic diagram would be that depicted in the inset of Figure 2a, which depending on the material, may extend over time scales covering many orders of magnitude. However, the time interval of the creep tests shown in Figure 2a,b cover about three orders of magnitude, between 10 and 3000 s. This is why only the linear part of the curve is observed. A measure of the viscoelasticity of the sample is the slope of this curve which we will call ‘r_D_’, as we will discuss below in Section 4. 

The PEM matrix exhibits the highest level of viscoelasticity, even though it is less deformable than other ECM-based materials. Interestingly, the values of ‘r_D_’ show no significant difference between the longitudinal and transversal directions, despite substantial disparities in compliance values (see Figure 2c). Once the stress applied in the creep test is removed, the sample tends to recover its original length. The evolution of compliance over time in the recovery process is shown in Figure 2d. The recovery process is characterized by a distinctive initial curvature in the double logarithmic diagram, but we are unable to extrapolate the precise duration required for full recovery from the initial deformation, as observed during the loading process. Notably, PEM-D displays a greater propensity for recovery from the initial deformation compared to other matrices, possibly owing to its increased thickness and the perpendicular alignment of collagen fibers (Figure 2d).

**Figure 3:**
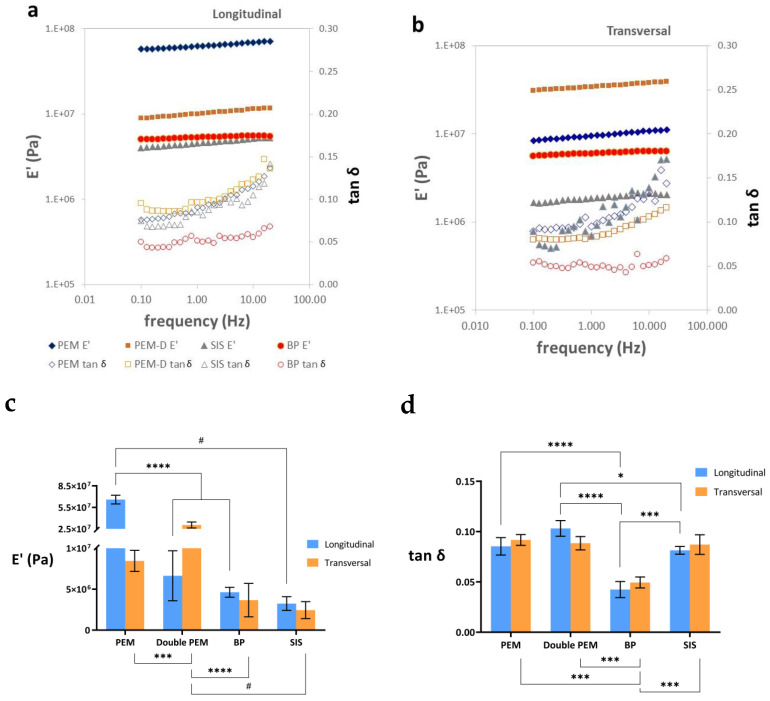
Results of the dynamic-mechanical tests in tension on PEM, or two, PEM-D, layers small intestine submucosa, SIS, and bovine pericardium, BP. Frequency dependence of the real part of the elastic modulus and tan δ measured in (**a**) the longitudinal direction and (**b**) the transversal one. (**c**) E′ measured at 1 Hz and (**d**) tan δ measured at 1 Hz of the four types of matrices in their longitudinal and transversal sections. All the bar diagrams represent the mean value ± standard deviation. The level of statistical significance is shown by the following legend: *p* ≤ 0.05 (*), *p* ≤ 0.001 (***), *p* ≤ 0.0001 (****) in normal samples and *p* ≤ 0.05 (#) in non-normal ones.

The results of dynamic-mechanical tests conducted at frequencies ranging from 0.1 to 20 Hz in both the longitudinal and transversal directions under tensile loading conditions are depicted in Figure 3. The situation is analogous to what we have described for the creep test above. The experimental frequency interval is not sufficient to show the sigmoid shape of the curve of the real part of the elastic modulus E′ and presents a linear relationship in the double logarithmic scale diagram (Figure 3a,b). Similarly, the loss tangent tan δ increases with increasing frequency but does not reach its characteristic maximum.

When assessing the tan δ, in the longitudinal orientation, PEM-D exhibits higher viscosity, followed by PEM. However, upon comparing the transversal orientation, PEM and SIS display a more pronounced viscous component than PEM. This observation could potentially be attributed to the alignment of collagen fibers and other proteins within the matrix. In the case of BP, its behavior in both directions appears isotropic, and lower than other EMC (Figure 3c,d). 

**Figure 4:**
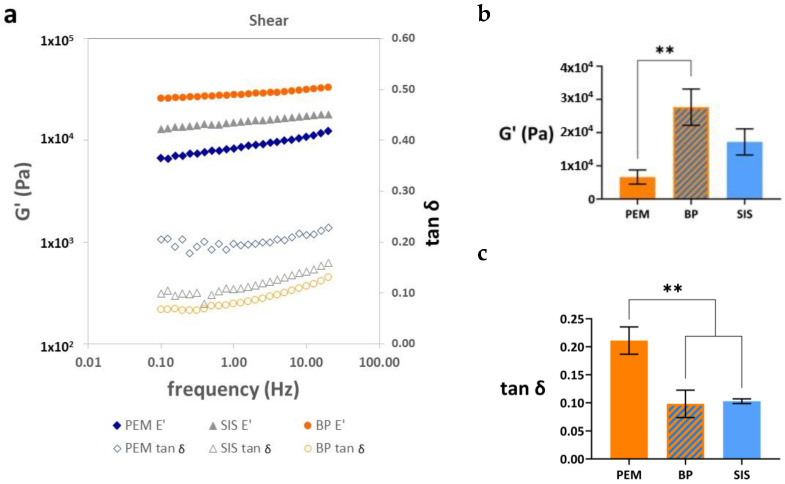
Results of the dynamic-mechanical tests in shear on PEM, small intestine submucosa, SIS, and bovine pericardium, BP. (**a**) Frequency dependence of the real part of the elastic modulus and the values of tan δ, (**b**) G′ measured at 1 Hz, (**c**) tan δ measured at 1 Hz. All the bar diagrams represent mean value ± standard deviation. The level of statistical significance is shown by the following legend: *p* ≤ 0.01 (**).

The tests performed in shear in the parallel plate rheometer are shown in Figure 4. The plots of G’ and tan δ show a similar shape to that of E’ and tan δ measured in the tension mode. The shear deformation mode provides information on the sliding of some fabric fibers over others. The PEM decellularized tissue shows more deformability than SIS or BP. 

Based on the preceding results, the key differences among BP, SIS, PEM, and PEM-D in terms of viscoelasticity and recovery can be summarized. In terms of viscoelasticity, PEM exhibits the highest overall viscoelasticity despite having lower initial deformability, indicating higher stiffness. SIS demonstrates moderate viscoelasticity with similar compliance values in both directions. PEM-D shows similar viscoelasticity to PEM, but is potentially slightly lower due to reduced initial compliance, while BP has the lowest viscoelasticity, suggesting minimal energy storage and dissipation.

## 4. Discussion

To regenerate tissues by tissue engineering techniques, a three-dimensional support seeded with cells can be implanted at the site of the damage, or alternatively, in cell-free strategies, the implanted support can be expected to be invaded by cells from its neighboring environment. In both cases, the support generates a biomechanical environment in which the material provides the cells with attachment sites, guides the organization of the newly formed extracellular matrix (ECM), and transmits mechanical stress to the cells. These stresses are key factors in determining the cells’ phenotype and therefore their ability to generate functional tissue through mecanotransduction signaling. In experiments carried out on mesenchymal stem cells (MSCs), it was found that the mechanical characteristics of the substrate on which these cells were seeded had an important influence on their differentiation: in very stiff matrices, the MSCs acquired an osteogenic phenotype, while in softer matrices, they could differentiate into chondrocytes or other cells of mesenchymal lineage [30], so that it is important to understand the mechanical behavior of 3D supports for their use in regenerative medicine [31,32,33,34,35]. While traditional mechanical testing often focuses on rupture behavior, this paper argues that evaluating the viscoelastic properties of acellular tissues is of paramount importance. The viscoelastic properties provide a more comprehensive and realistic representation of how these tissues respond to external forces, over rupture behavior alone. This paper discusses the rationale behind this perspective, highlighting its relevance in fields such as biomaterials science, regenerative medicine, and biomechanics.

In the tensile creep tests, it was found that the simple PEM porcine esophageal-derived matrices tested longitudinally had greater rigidity (lower compliance values) than the SIS matrix and much more so than the BP matrix, which is the most deformable, although the double PEM sample showed greater compliance than the monolayer. This effect can be attributed to one layer sliding over another, showing that the inter-layer adhesion of the decellularized matrix is weaker than the structure of the fibers aligned inside each layer, as can be expected, since the two layers are only held together by the physical interaction caused by freezing and drying.

In the case of the PEM matrix, the compliance value is three times greater in the transversal than in the longitudinal direction, while in SIS and BP, it is approximately twice as high. In this direction, the PEM matrix behaves similarly to the SIS, while the BP is once again the most deformable, with much higher compliance values than the others. Interestingly, the double-PEM matrix presents strain values in the transverse direction of the same order as in the longitudinal, showing that the orientation of the fiber bundles in one layer perpendicular to another stiffens the assembly in spite of inter-layer sliding.

It should be noted that the characteristic sigmoid shape is not found in the curves in Figure 2, as has been reported in other decellularized tissues [24,25,27,28]. Many viscoelastic or dielectric relaxation processes can be accurately reproduced by the Kohlraush–Williams–Watts Function (KWW) [36] or the stretched exponential equation. The compliance measured in tension as a function of time D(t) in a creep test KWW equation can be written as follows [37]:

(1) $$\varphi \left(t\right)=\frac{D\left(t\right)-D_{U}}{D_{R}-D_{U}}=1-exp\left[-{\left(\frac{t}{\tau }\right)}^{\beta }\right]$$

where *φ*(*t*) is the relaxation function, which varies between 0 and 1, *D_U_* and *D_R_* are the limit values of *D*(*t*) at time 0 and infinite time, respectively, (unrelaxed and relaxed compliance, respectively), and *β* is a coefficient that varies between 0 and 1, (for *β* = 1 it corresponds to the single relaxation time model) and *τ* can be called the relaxation time. The shape of this curve for *β* = 0.5 is represented in Figure 2a, with arbitrary values for the rest of the parameters. It can be seen that the relaxation process is extremely wide, which justifies the use of the logarithmic scale on the time axis and also that the relationship between log *D*(*t*) and log *t* is approximately a straight line. 

The experimental results in our time interval, which covers around three time decades, show an approximately linear relationship that does not allow any form of extrapolation in order to determine the *D_U_*, *D_R_*, *β* parameters or the *τ* of Equation (1), although alternative parameters can be equally useful to characterize the viscoelastic response. What the cells seeded in a viscoelastic environment such as these biological matrices can detect is actually the magnitude of the deformation of the matrix to which they adhere, i.e., their rigidity and the speed at which this deformation occurs under the force that the cell or the environment can exert on the tissue. It may thus be sufficient to use the slope of the curve shown in Figure 2 as a parameter to characterize the viscoelastic response, which we can call the response speed in compliance (r_D_). Analyzing the data based on this response speed instead of the relaxation times or relaxation intensities avoids making extrapolations that can lead to huge interpretation errors.

Figure 2c shows that the PEM matrix shows the greatest viscoelasticity even though it is the least deformable. When the load is applied, the tissue is initially stiffer but gradually yields. To our knowledge, it is not known how this more viscoelastic behavior affects cellular responses. It is interesting that the values of r_D_ are not significantly different in the longitudinal and transversal directions, in spite of significant differences in the value of the compliance.

The recovery after removing the applied load is shown in Figure 2d, again using the double logarithmic scale. The characteristic initial curvature of the relaxation process can be seen here, although the experimental times do not allow us to venture an extrapolation of the time necessary to reach complete recovery from the initial deformation, as occurred in the loading process. The recoverable deformation of the extracellular matrix depends not only on the elastic characteristics of the organ or tissue from which the ECM is extracted, but also on the methods used to obtain it.

In the dynamic-mechanical tests in tension, the real part of the elastic modulus with frequency can be seen to increase. Figure 3a,b show this behavior in the longitudinal and transversal direction tests, respectively. As in the creep measurements, it is clear that the viscoelastic relaxation process is much broader than the three decades of frequency available in the equipment used (between 0.1 and 20 Hz). The tan δ values increase with the logarithm of frequency, but they are also far from the characteristic peak of the relaxation process. We used the values of E′ and tan δ measured at a frequency of 1 Hz to compare the stiffness of the different samples (Figure 3b,c, respectively).

When evaluating the storage modulus (Figure 3b), differences were found in the properties of the different matrices and in terms of the orientation of the samples at the time of the tests. The simple PEM in the longitudinal direction was found to be more rigid than the other samples evaluated, in line with the results of the creep tests. Also, the measurement in the transverse direction gives a much lower E′ value, in good agreement with the creep tests. The greatest stiffness shown in the creep experiments in the double-PEM matrix in the transverse direction is confirmed, with values close to the measurement of the PEM matrix in the longitudinal direction, probably due to the recruitment of the longitudinal fibers provided by the overlapping fibers. However, this behavior is not found when evaluating the double-PEM sample in the longitudinal direction, since it shows similar behavior to the PEM sample in the transverse direction. No significant differences were found in the SIS and BP samples between the longitudinal and cross-sectional direction measurements. The fact that the sample is subjected to smaller deformations in the dynamic-mechanical experiments than in creep reduces the influence of the orientation of the collagen and elastin fibers on the elastic modulus measured.

The parallel plate rheometer test is a measure of shear strength (see results in Figure 4). They are qualitatively analogous to those obtained in the tension measurements: a small increase in G′ with frequency, with a linear dependence of log G′ on log f and the curvature of the dependence of tan δ on log f, but far from reaching a maximum. However, all the matrices presented a shear modulus more than two orders of magnitude lower than the Young’s modulus measured in tension. In shear, we measured the deformation produced by some fiber layers sliding over others, and it seems clear that the resistance to this type of stress is weak in this type of tissue, as expected. The value of tan δ, which to a certain extent measures the internal friction in the material, was somewhat higher, but in the same order of magnitude as that measured in tension.

Significant differences were found in terms of shear strength, which depended on the origin of the matrices, while the bilayer double-PEM derived from the porcine esophagus had the highest shear modulus, followed by the SIS and the BP, PEM tissue with lower shear strength.

In this comparative biomechanical characterization assay, it was found that the matrices from the porcine esophagus had greater rigidity than those of porcine intestinal submucosa and bovine pericardium in the evaluation of the longitudinally oriented samples. However, when these were evaluated in the transverse direction, the bovine pericardium was found to be more rigid than the intestinal submucosa and the esophageal matrix in the creep test.

These results can be partially explained by the structure and function of the organs from which matrices were obtained: the esophagus serves a transit function and allows the bolus to pass from mouth to stomach, so that it has to relax transversely by up to three times its size, while it is virtually immobile in the longitudinal direction. The functions of the small intestine are mainly absorptive and propulsive: it distends in all directions despite the fact that in vivo, it has little tolerance to increasing pressure, while by contrast the pericardium is a fibrous membrane with nonelastic properties that limit acute cardiac dilatation. It supports cyclic cardiac distension and contraction at a minimum of 60 times per minute, while resisting the surrounding forces derived from the breathing cycles, which modify the pressure inside the thoracic cavity so that their fibers are intertwined. However, under normal physiological conditions, unlike the esophagus, both the small intestine and the pericardium are subject in vivo to low pressures and also have a more fluid behavior than the esophagus [38,39,40,41,42,43,44,45].

The stiffness of the matrices derived from porcine esophagus was found to decrease more than the SIS and pericardium meshes, probably due to the reorganization and redistribution of the forces inside the matrix collagen fibers.

The comprehensive search in various scientific databases for information on the viscoelastic properties of extracellular matrices derived from bovine pericardium, porcine esophagus, and small intestine submucosa has unfortunately yielded results indicating a scarcity of available information in the scientific literature regarding these specific materials.

Most of the published works on the mechanical evaluations on biological matrices have reported on the response measured in tension. The studies by Badylak et al. found that the acellular small intestine submucosa matrices, in a push-through failure load test, had a value of 433.6 +/− 79.5 N, which was higher than that reported by Arnold et al. [46], who reported a maximum load for this material of 26.65 +/− 7 N. This difference could be attributed to potential variations in the production methods of the different matrices.

In the study published by Chaitin et al. [47], they demonstrate that the decellularization process preserves the mechanical integrity and extracellular matrix composition of esophageal tissue. In that study, it was observed that native esophageal mucosa exhibited a maximum extension stress between 1.5 and 2.0 MPa at around 450% extension, while the decellularized tissue showed a maximum extension stress between 4.0 MPa and 4.5 MPa at 600% extension. However, there were no significant differences in the elastic modulus, which was 19 +/− 20 and 20 +/− 8 MPa, respectively, which are lower than the values reported in this work both in the longitudinal and transversal direction.

Regarding bovine pericardium, Hülsmann et al. [48] reported an elastic modulus for native tissue of 27 +/− 6 MPa, and after decellularization, changes in the elastic modulus were observed, depending on the method used, ranging between 10 and 6 MPa. This variation might be attributed to the aldehydes used in the tissue preservation process. It is worth noting that no studies were found that delved deeper into the viscoelastic response of these tissues.

Despite the importance of understanding the viscoelastic properties of ECMs in biomedical and tissue engineering applications, it appears that specific research on these particular tissues has not been extensively documented in the scientific community. This may be due to their relative rarity or a more limited focus compared to other biologically derived materials.

Given the potential significance of such research for medical and engineering applications, there may be a need for further efforts in generating data and studies related to the viscoelastic properties of ECMs derived from bovine pericardium, porcine esophagus, and small intestine submucosa. This gap in the literature could represent an opportunity for future research and scientific contributions that enrich our understanding of these materials and their diverse applications.

## 5. Conclusions

In this work, we evaluated the mechanical properties of decellularized matrices obtained from the porcine esophagus, formed either by one or two layers of tissue using two commercial matrices of biological origin as a reference.

The decellularization of porcine esophageal tissue allows acellular matrices to be obtained with a higher elastic modulus than those of the two commercial biological tissues used as controls while showing a marked viscoelastic behavior with a greater time or frequency dependence on the mechanical properties than the other matrices. The compliance measured in tension increases significantly over time after the gradual application of a stress. This behavior is consistent with the tissue’s microstructure, which presents longitudinally aligned fibers, and with the function of the in vivo tissue. The stacking of two layers of fabric did not lead to a higher tensile strength, possibly due to slippage at the interface. However, the acellular tissue derived from the porcine esophagus was more prone to deform than the controls in both transverse tension and shear, which also correlated with the orientation of the fiber bundles. In these loading modes, the stacking of two layers of fabric did lead to a clear increase in the mechanical resistance.

## Data Availability

Publicly available datasets were analyzed in this study. These data can be found at: Riunet.upv.es.

## Supplementary Materials

The following supporting information can be downloaded at: https://www.mdpi.com/article/10.3390/ma17010134/s1, Figure S1: Nuclear content (A) before and (B) after the decellularization process by quantification in the cellprofiler software, version 3.5.2.
