# Supplementary material for: Viscoelastic Properties of Acellular Matrices of Porcine Esophageal Mucosa and Comparison with Acellular Matrices of Porcine Small Intestine Submucosa and Bovine Pericardium
Source: Materials (Basel). 2023 Dec 27;17(1):134. doi: 10.3390/ma17010134 (PMC10779732; doi:10.3390/ma17010134)
Supplement: Supplementary file 1 [file materials-17-00134-s001.zip › materials-2669880-supplementary.pdf]

materials

Supplementary

S1.

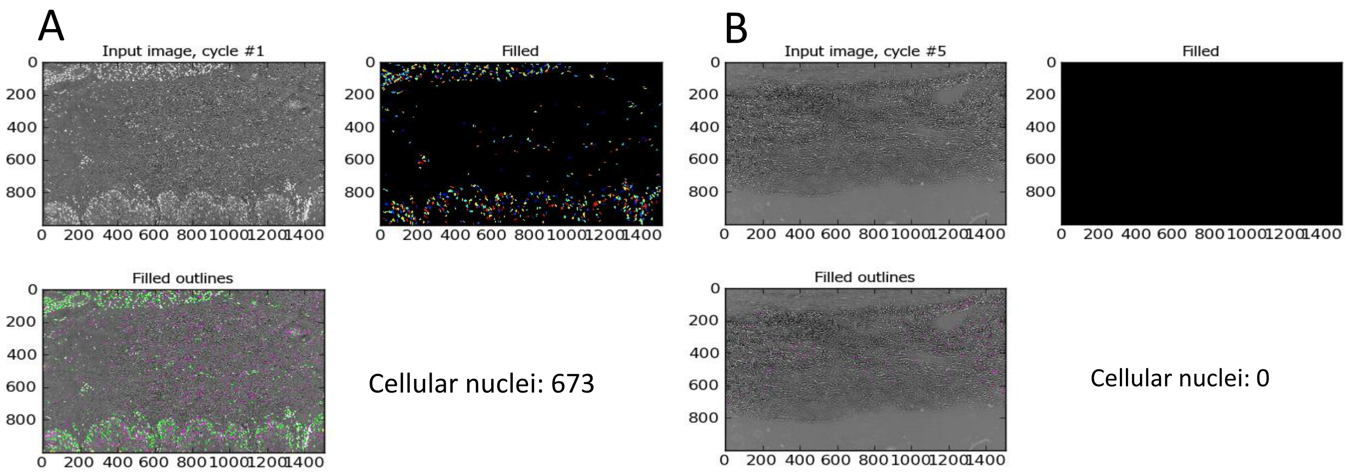

Figure S1. Nuclear content (A) before and (B) after the decellularization process by quantification in the cellprofiler software, version 3.5.2.
